# Prediction Model for Discharge Destination in Older Patients After Hip Fracture Surgery Using Early Predictors

**DOI:** 10.3390/geriatrics11050130

**Published:** 2026-09-11

**Authors:** Sanne M. Krakers, Dieuwke van Dartel, Marloes Vermeer, Miriam M. R. Vollenbroek-Hutten, Johannes H. Hegeman

**Affiliations:** 1 Biomedical Signals and Systems Group, University of Twente, 7500 AE Enschede, The Netherlands; 2 Department of Trauma Surgery, Ziekenhuisgroep Twente, 7609 PP Almelo, The Netherlands; 3 ZGT Academy, Ziekenhuisgroep Twente, 7609 PP Almelo, The Netherlands; 4 Medisch Spectrum Twente, 7512 KZ Enschede, The Netherlands

**Keywords:** discharge destination, geriatric rehabilitation, hip fracture, older patients, predictors

## Abstract

**Objectives**: To identify early predictors of discharge destination in patients aged ≥70 years following hip fracture surgery, using the Dutch Hip Fracture Audit (DHFA) Indicator Taskforce database with the aim of developing and validating a prediction model. **Methods**: Patients living in a (residential) home prior to the hip fracture were categorized into three discharge groups: home (*n* = 1322), geriatric rehabilitation (*n* = 2181), and nursing home (*n* = 337). The dataset was randomly split into a training set (75%) and a test set (25%). Potential predictors of discharge destination were collected immediately after hip fracture surgery and first tested univariately in the training set. Variables showing *p* < 0.15 were entered into a multinomial regression model. The model was validated on the test set. **Results**: The training set consisted of 2878 patients (median age: 83 years (IQR 77–89 years), 2012 females (70%)). The final multinomial regression model included age, gender, PFMS, premorbid Katz-ADL, ASA score, polypharmacy, history of dementia, delirium during previous hospital admissions, fall in the past six months, surgical treatment, anaesthesia type, and co-treatment by a geriatrician. The strength and direction of the associations between these variables and discharge destination varied across the three pairwise comparisons. The validated model predicted discharge to geriatric rehabilitation, home, and a nursing home with sensitivities of 86.7%, 37.9%, and 9.6%, respectively. Precision ranged from 33.3% (nursing home) to 60.9% (home) and 66.7% (geriatric rehabilitation). **Conclusions**: Discharge destination could be predicted with relatively high sensitivity and moderate precision for geriatric rehabilitation, whereas sensitivity and precision for predicting discharge home and to a nursing home remained limited. This highlights the need to refine the model before clinical implementation can be considered.

## 1. Introduction

In the Netherlands, more than 22,000 patients were hospitalized with a hip fracture in 2023, most of whom were aged ≥70 years [1]. Dutch hip fracture care is guided by a national guideline, emphasizing timely surgery and multidisciplinary treatment [2]. Recent nationwide data indicate that 64.9% of patients undergo surgery within 24 h and have a median hospital length of stay of approximately six days [3]. After hospitalization, approximately half of the patients are temporarily admitted to a skilled nursing home for geriatric rehabilitation to support further recovery following hip fracture surgery [4,5]. Discharge planning in the hospital is logistically challenging. The appropriate discharge destination often remains uncertain in the early postoperative phase. Decisions depend on the patient’s functional recovery, medical stability, and cognitive status. As a result, discharge planning is frequently initiated a few days after hip fracture surgery. Subsequently, a request for geriatric rehabilitation must be submitted to the hospital discharge planning coordinator, who evaluates the application and obtains approval from an elderly care physician. After approval, an available rehabilitation facility must be identified, which also takes time and may lead to prolonged hospitalization. Van den Ende et al. showed that 21% of patients in three Dutch hospitals experienced discharge delays, with a median length of stay twice as long as that of patients without delay, and that 74% of these delays were caused by factors outside the hospital, most commonly waiting for a place in care homes [6]. Prolonged hospital stays are associated with slower recovery, increased risk of complications, higher mortality rates, and increased healthcare costs [7,8,9,10,11]. Early prediction of a patient’s discharge destination immediately after hip fracture surgery could enhance discharge planning and help prevent unnecessary delays in hospital discharge.

Van Dartel et al. identified age, Pre-Fracture Mobility Score (PFMS), premorbid Katz Index of Independence in Activities of Daily Living (Katz-ADL), history of dementia, American Society of Anaesthesiologists physical status classification (ASA) score, type of anaesthesia, surgical treatment, and co-treatment by a geriatrician as independent early predictors for discharge to geriatric rehabilitation in older patients after hip fracture surgery using data derived from the Dutch Hip Fracture Audit (DHFA), the national hip fracture registry [4]. However, the developed multinomial regression model correctly predicted only 86%, 38.6%, and 2.4% of patients discharged to geriatric rehabilitation, home, and a nursing home, respectively. Furthermore, the model was not validated, limiting its clinical applicability. In line with these findings, a few recent studies have identified predictors of discharge to geriatric rehabilitation or inpatient care facilities (skilled nursing or rehabilitation facility), including: age [12,13,14], premorbid Katz-ADL (premorbid functional health status) [12,13], ASA score [13,14], history of dementia [12], surgical treatment [12], anaesthesia type [13], multiple comorbidities (diabetes [12,13], congestive heart failure [12], chronic renal failure [12], history of cerebrovascular accident [12], hypertension [12,13], chronic obstructive pulmonary disease [12,13], baseline anticoagulation therapy [12], and history of myocardial infarction [12]), higher Body Mass Index (BMI) [13], intertrochanteric hip fractures [12], longer hospital stay [12,13], and in-hospital complications [12,13]. While these studies highlight relevant predictors, most focused on broader inpatient care facilities rather than specifically on geriatric rehabilitation, or did not develop and validate prediction models suitable for early clinical decision-making. Consequently, there remains a need for an optimized and validated prediction model that supports discharge planning immediately after hip fracture surgery in older patients.

In 2017, the DHFA Indicator Taskforce was established to optimize data collection quality and identify and evaluate new variables, with the aim of developing future quality indicators to support further improvement and benchmarking of hip fracture care. As part of this initiative, seven hospitals collected an extended dataset including additional variables such as serum haemoglobin level at hospital admission, polypharmacy (use of more than five medications before admission), preoperative dose of tranexamic acid, the Parker Mobility Score (PMS) at hospital admission, memory problems, delirium during previous hospital admissions, and a fall in the past six months. The availability of these additional variables, which have not previously been examined in the context of early discharge destination prediction after hip fracture surgery, provides an opportunity to examine whether incorporating them improves predictive performance. Therefore, the aim of this study is to identify early predictors of discharge destination in patients aged ≥70 years following hip fracture surgery using the more comprehensive DHFA Indicator Taskforce database, in order to develop and validate an improved prediction model.

## 2. Materials and Methods

### 2.1. Study Population

**Figure 1:**
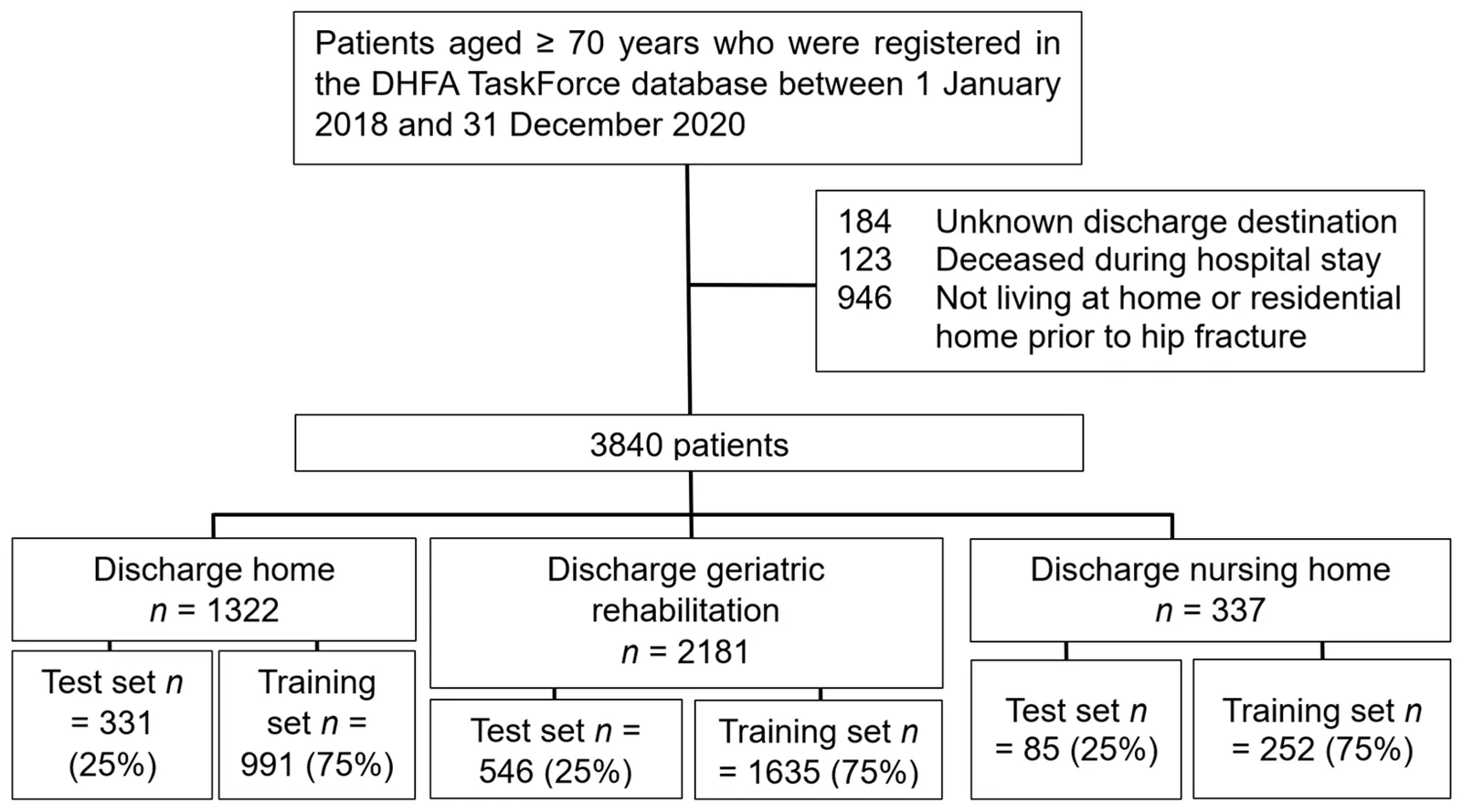
Flowchart of study design.

Data from hip fracture patients aged ≥70 years who underwent surgical treatment between 1 January 2018 and 31 December 2020 and were registered in the DHFA Indicator Taskforce database were used in this study. Exclusion criteria for this study were an unknown discharge destination, in-hospital mortality, and not living at (residential) home prior to the hip fracture (Figure 1). Patients were categorized into 3 groups based on their discharge destination: discharge home, discharge to geriatric rehabilitation, and discharge to a nursing home.

### 2.2. Data Collection

The following baseline variables were collected from the DHFA Indicator Taskforce database: age; gender; PFMS; PMS; premorbid Katz-ADL; ASA score; usage of osteoporosis medication; usage of direct oral anticoagulants (DOAC); polypharmacy; history of dementia; memory problems; delirium during previous hospital admissions; fall in the past six months; nutritional status and perioperative variables (serum haemoglobin level upon hospital admission; preoperative dose of tranexamic acid; fracture type; surgical treatment; type of anaesthesia; co-treatment by a geriatrician). The PFMS assesses walking independence and ranges from 1 (freely mobile without aids) to 5 (no functional mobility) [15]. Patients were regrouped into three groups depending on their score: fully mobile (1), mobile with aids (2–3), and indoor confined (4–5). The ASA score measures the patient’s condition prior to surgery, ranging from 1 (normal healthy patient) to 5 (moribund patient) [16]. Patients were regrouped into two groups based on their score: ASA 1–2 and ASA 3–5. The DHFA Indicator Taskforce database covered two types of scoring with regard to the nutritional status: the Short Nutritional Assessment Questionnaire (ranges from 0 to 7, ≥3 indicating malnutrition) and the Malnutrition Universal Screening Tool (ranges from 0 to 6, ≥2 indicating malnutrition) [17]. The SNAQ or MUST was used depending on the participating hospital in which the patient was treated. As both are validated nutritional screening tools with comparable performance in identifying nutrition risk [17], each score was dichotomized according to its established cut-off. The resulting classifications were combined into a binary variable indicating the presence or absence of malnutrition. Variables with a substantial proportion of missing data were excluded prior to analysis.

### 2.3. Statistical Analysis

Continuous variables were described as mean with standard deviation (SD) in case of a normal distribution or as median with interquartile range (IQR) in case of a skewed distribution. Categorical variables were presented as numbers with the corresponding percentage. The dataset was split into a training set (75% of patients) and a test set (25% of patients) (Figure 1). The percentage of missing values per variable in the training set ranged from 0% to 46.4%. Data completeness for the variables in the training set was 92% overall.

### 2.4. Model Training

The association between the variables and discharge destination was first tested univariately on the training set, using a one-way analysis of variance (ANOVA) or a Kruskal–Wallis test for continuous variables (depending on the distribution of the data), and a Chi-square test for categorical variables. Missing data were not imputed. For each analysis, patients with missing data on variables required for that analysis were excluded. Variables with a *p*-value < 0.15 were entered into a backward stepwise multinomial regression model. A complete-case analysis was applied, whereby only patients with complete data for all candidate predictor variables were included. In the model, variables not reaching statistical significance were eliminated from the model in a stepwise manner, starting with the variable with the highest *p*-value, until all remaining variables were statistically significant (*p*-value < 0.05). All analyses were performed using SPSS version 25.0 (IBM Corp., Armonk, NY, USA).

### 2.5. Model Validation

Model validation was performed in R (version 4.4.2) using the test set. The same complete-case analysis approach used during model training was applied, excluding patients with missing values in any of the predictor variables included in the final model. For each patient in the test set, the model predicted a probability for each discharge destination. Based on these probabilities, the area under the receiver operating characteristic curve (AUC) was calculated and a calibration plot was generated for each discharge destination. The destination with the highest probability was assigned as the predicted discharge destination. The performance of the model was evaluated on the test set using the classification table and several performance metrics. Sensitivity (the proportion of correctly predicted patients within a specific discharge destination), specificity (the proportion of patients correctly predicted as not belonging to a specific discharge destination), precision (the proportion of correct classifications among all predictions for that discharge destination), prevalence (the proportion of patients in the dataset who actually belong to a specific discharge destination), detection prevalence (the proportion of patients predicted to belong to a specific discharge destination, regardless of the observed discharge destination), and balanced accuracy (the average of sensitivity and specificity per discharge destination) were calculated separately for each discharge destination. Overall accuracy was defined as the proportion of correctly classified patients across all discharge destinations.

## 3. Results

### 3.1. Study Population

From 1 January 2018 to 31 December 2020, a total of 5095 patients underwent hip fracture surgery and were registered in the DHFA Indicator Taskforce database. Of those patients, 184 had an unknown discharge destination, 123 died during hospital stay, and 946 were not living at home or in a residential home prior to the hip fracture and were excluded, leaving 3840 patients in this study (2878 in the training set and 962 in the test set). Table 1 presents the baseline and perioperative characteristics of the training set. PMS (number missing = 1334), usage of DOAC (number missing = 1037) and preoperative dose of tranexamic acid (number missing = 609) were excluded from the analysis due to a substantial proportion of missing data. Fracture type was also excluded, as the variable was strongly associated with the type of surgical treatment. Surgical treatment was considered more patient-specific, depending not only on the type of fracture but also on the patient’s condition.

### 3.2. Model Training

Age, gender, PFMS, Premorbid Katz-ADL, ASA score, usage of osteoporosis medication, polypharmacy, history of dementia, memory problems, delirium during previous hospital admission, fall in the past six months, malnutrition, serum Hemoglobin level, surgical treatment, type of anaesthesia, and co-treatment by a geriatrician were included in the multinomial regression analysis (Table 1).

The final multinomial regression model was developed using data from 2227 patients with a complete dataset on all candidate predictor variables. This final model included the following variables: age, gender, PFMS, premorbid Katz-ADL, ASA score, polypharmacy, history of dementia, delirium during previous hospital admission, fall in past six months, surgical treatment, anaesthesia type, and co-treatment geriatrician (Table 2). The strength and direction of the associations between these variables and discharge destination varied across the three pairwise comparisons.

Independent predictors of discharge to geriatric rehabilitation vs. home included higher age (OR = 1.1, 95% CI 1.0–1.1), mobile with aids (PFMS 2–3) (OR = 1.8, 95% CI 1.4–2.2) and indoor confined (PFMS 4–5) (OR = 2.1, 95% CI 1.5–3.0) vs. fully mobile (PFMS 1), ASA score 3–4 vs. 1–2 (OR = 1.4, 95% CI 1.1–1.7), polypharmacy (OR = 1.5, 95% CI 1.2–1.9), a fall in the past 6 months (OR = 1.5, 95% CI 1.2–1.8), intramedullary implant vs. hemiarthroplasty (OR = 1.6, 95% CI 1.3–2.0), and cotreatment by a geriatrician (OR = 1.4, 95% CI 1.0–1.8) (Table 2). In contrast, being male vs. female (OR = 0.8, 95% CI 0.6–1.0), history of dementia (OR = 0.6, 95% CI 0.4–0.8), delirium during previous hospital admissions (OR = 0.7, 95% CI 0.5–0.9), and total hip arthroplasty vs. hemiarthroplasty (OR = 0.4, 95% CI 0.2–0.7) reduced the likelihood of being discharged to geriatric rehabilitation (vs. home).

Independent predictors of discharge to geriatric rehabilitation vs. nursing home included spinal vs. general anaesthesia (OR = 1.9, 95% CI 1.3–2.7) (Table 2). Higher premorbid Katz-ADL (OR = 0.8, 95% CI 0.7–0.9), history of dementia (OR = 0.3, 95% CI 0.2–0.4), delirium in previous admissions (OR = 0.6, 95% CI 0.4–0.9), and cotreatment by a geriatrician (OR = 0.5, 95% CI 0.2–0.9) decreased the likelihood of discharge to geriatric rehabilitation (vs. nursing home).

Independent predictors of discharge to nursing home vs. home included higher age (OR = 1.1, 95% CI 1.0–1.1), mobile with aids (PFMS 2–3) (OR = 2.1, 95% CI 1.3–3.2) and indoor confined (PFMS 4–5) (OR = 2.2, 95% CI 1.2–4.0) vs. fully mobile (PFMS 1), higher premorbid Katz-ADL (OR = 1.2, 95% CI 1.1–1.3), polypharmacy (OR = 2.1, 95% CI 1.4–3.1), history of dementia (OR = 2.0, 95% CI 1.2–3.2), a fall in the past 6 months (OR = 1.6, 95% CI 1.1–2.5), and cotreatment by a geriatrician (OR = 2.9, 95% CI 1.4–5.7). Spinal vs. general anaesthesia (OR = 0.5, 95% CI 0.3–0.7) was associated with a decreased likelihood of discharge to a nursing home (vs. home).

### 3.3. Model Validation

**Figure 2:**
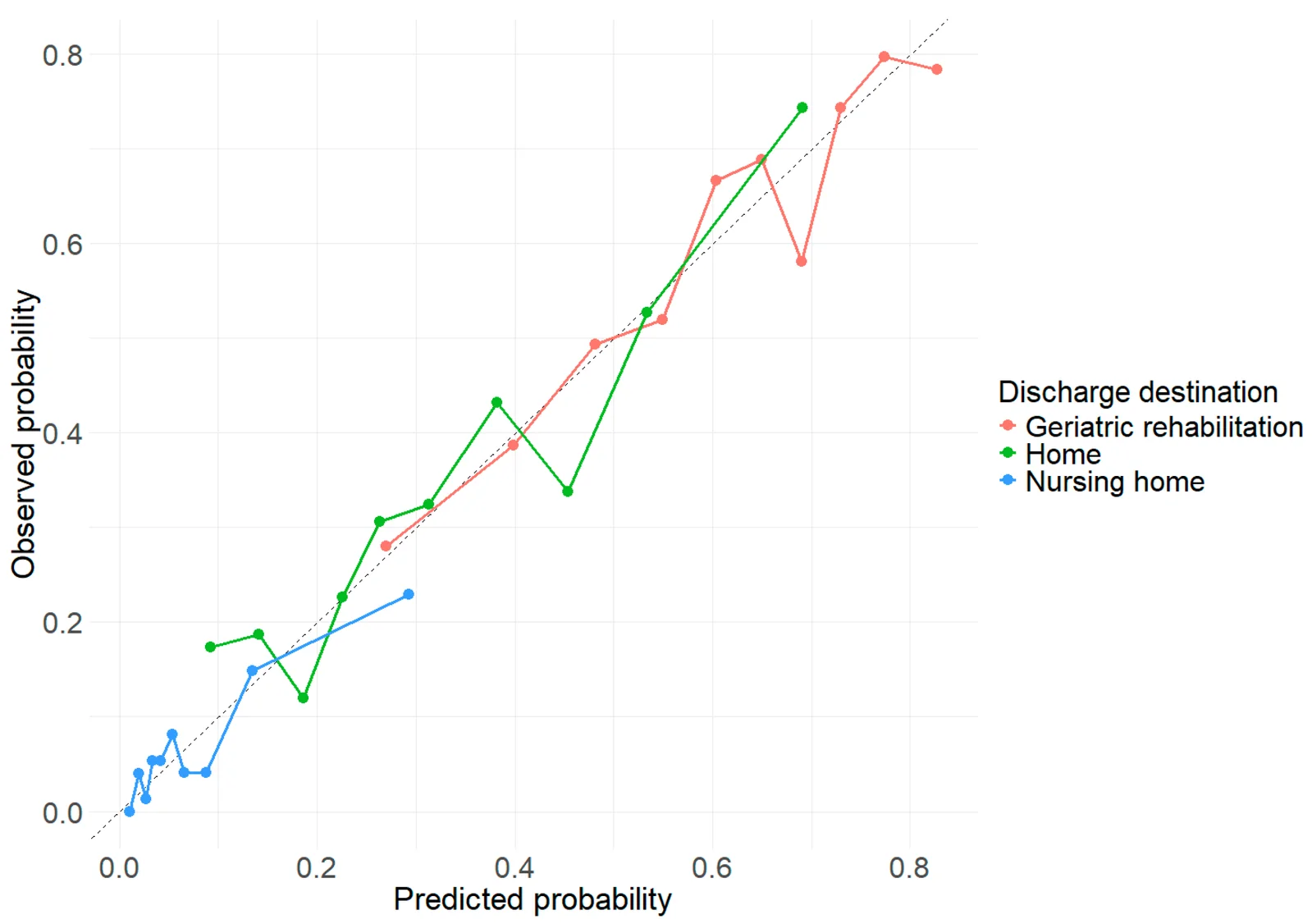
Calibration plots by discharge destination.

Calibration plots for the three discharge destinations of the test set are presented in Figure 2. Overall, the model showed reasonable agreement between predicted and observed probabilities. The AUC was 0.708 (95% CI 0.668–0.748) for discharge home, 0.690 (95% CI 0.651–0.728) for discharge to geriatric rehabilitation, and 0.737 (95% CI 0.667–0.807) for discharge to a nursing home (Table 3).

Furthermore, Table 3 presents the classification table and performance of the prediction model on the test set. Model performance varied across discharge destinations. Sensitivity was high for the rehabilitation group (86.7% (95% CI 83.2–89.5)) but low for the home (37.9% (95% CI 32.1–44.0)) and nursing home groups (9.6% (95% CI 4.2–20.6)). Specificity was highest for the nursing home group (98.6% (95% CI 97.4–99.2)) and lowest for the rehabilitation group (37.0% (95% CI 31.7–42.5)). Precision ranged from 33.3% (95% CI 15.2–58.2) (nursing home group) to 66.7% (95% CI 62.8–70.5) (rehabilitation group). The prevalence and detection prevalence were 33.7% (95% CI 30.4–37.2) and 20.9% (95% CI 18.2–24.0) for the home group, 59.3% (95% CI 55.8–62.8) and 77.1% (95% CI 73.9–79.9) for het rehabilitation group, and 7.0% (95% CI 5.4–9.0) and 2.0% (95% CI 1.2–3.3) for the nursing home group. Overall model accuracy was 64.8% (95% CI 61.3–68.3), with balanced accuracies of 62.8% (95% CI 14.3–94.5), 61.8% (95% CI 13.9–94.2), and 55.1% (95% CI 10.9–91.9) for the home, rehabilitation, and nursing home groups, respectively.

## 4. Discussion

The aim of this study was to identify early predictors of discharge destination in patients aged ≥70 years following hip fracture surgery and to develop and validate an improved prediction model. Unlike the previous study described by Van Dartel et al. [4], the current study used the more comprehensive DHFA Indicator Taskforce database. This database contains additional clinically relevant variables that have not previously been examined in relation to early discharge destination after hip fracture surgery, including polypharmacy, delirium during previous hospital admission, and a fall in the past six months. Furthermore, model development and testing were performed on separate training and test sets to reduce the risk of overestimation and overfitting. Results showed that age, gender, PFMS, ASA score, polypharmacy, history of dementia, delirium during previous hospital admissions, fall in the past six months, surgical treatment, and cotreatment by a geriatrician were independent predictors of discharge to geriatric rehabilitation vs. home. Premorbid Katz-ADL, history of dementia, delirium during previous hospital admissions, anaesthesia type, and cotreatment by a geriatrician were independent predictors of discharge to geriatric rehabilitation vs. nursing home. Age, PFMS, premorbid Katz-ADL, polypharmacy, history of dementia, fall in the past 6 months, anaesthesia type, and cotreatment by a geriatrician were independent predictors of discharge to nursing home vs. home. Discharge to geriatric rehabilitation, home, and a nursing home could be predicted immediately after hip fracture surgery with sensitivities of 86.7%, 37.9%, and 9.6%, respectively. This study showed only modest improvement in model discrimination compared to the study of Van Dartel et al. [4]. Sensitivity for discharge to geriatric rehabilitation increased slightly (86.7% vs. 86%), as did precision (66.7% vs. 63.5%). Sensitivity for discharge to a nursing home also increased (9.6% vs. 2.4%), while sensitivity for discharge home slightly decreased (37.9% vs. 38.6%). These small differences suggest that the inclusion of additional variables from the DHFA Indicator Taskforce database provided limited added discriminative value. However, because model development and testing were performed on separate training and test sets, the current study provides a more realistic estimate of model performance. Consequently, although performance gains were limited, the model is likely more robust and generalisable to new patient populations. For the early identification of patients requiring geriatric rehabilitation, achieving high sensitivity is important. Missing eligible patients can delay discharge planning and prolong hospital stays, thereby impairing recovery, increasing the risk of complications and mortality, and raising healthcare costs [7,8,9,10,11]. Early geriatric rehabilitation requests made immediately after hip fracture surgery can still be cancelled if clinical evaluation later suggests it is not needed. However, incorrectly classifying patients as requiring rehabilitation may result in temporary reservation of rehabilitation beds, thereby limiting availability for patients who truly need them. In this context, the model achieved relatively high sensitivity for the geriatric rehabilitation group (86.7%), indicating potential for timely discharge planning. However, precision was moderate (66.7%), suggesting the need for further optimization before clinical implementation.

The AUC values were calculated using the predicted probabilities for each discharge destination, suggesting poor to moderate discriminative performance with AUC values between 0.690 and 0.737. Despite an AUC of 0.737 for discharge to a nursing home, the sensitivity was low (9.6%). This indicates that the model was able to assign higher probabilities of nursing home discharge to patients who were actually discharged to a nursing home than those who were not, within the overall range of predicted nursing home probabilities. However, the model rarely classified them as discharged to a nursing home. Calibration plots demonstrated reasonable agreement between predicted and observed probabilities, indicating that the model provided reasonably accurate probability estimates across discharge destinations. These findings suggest that the prediction model may assist healthcare professionals in early hospital discharge planning by providing individualized probabilities for different discharge destinations. However, its limited discriminative performance indicates that the predictions should be interpreted cautiously. Therefore, the model should not be used as a stand-alone decision-making tool, but rather as an adjunct to clinical judgment.

Given the substantial class imbalance across discharge destinations and the small sample size of the nursing home discharge group, the performance of the prediction model should be interpreted with caution. As 59.3% of patients were discharged to geriatric rehabilitation, a naive baseline classifier always predicting geriatric rehabilitation would achieve an accuracy of 59.3%. The current prediction model achieved an overall accuracy of 64.8%, representing only a modest improvement over this baseline. These findings highlight the difficulty of accurately identifying patients discharged to destinations other than geriatric rehabilitation. Therefore, the current model does not demonstrate sufficient performance for clinical implementation.

In comparison with the predictors identified by Van Dartel et al. [4], the current study identified several additional independent predictors from the DHFA Indicator Taskforce database. Polypharmacy, delirium during previous hospital admissions, and a fall in the past six months were identified as additional predictors for discharge to geriatric rehabilitation (vs. home). Delirium during previous hospital admissions emerged as an additional independent predictor for discharge to geriatric rehabilitation (vs. nursing home). Polypharmacy and a fall in the past six months were identified as additional predictors for discharge to a nursing home (vs. home). Although the inclusion of these variables provided limited added discriminative value, they may contribute to a more comprehensive clinical characterisation of patients eligible for different discharge destinations. In contrast, some predictors found by Van Dartel et al. were not found to be significant in the present study [4]. Premorbid Katz-ADL and type of anaesthesia were not identified as independent predictors of discharge to geriatric rehabilitation (vs. home). ASA score and surgical treatment were not identified as independent predictors of discharge to a nursing home (vs. home). The use of the more comprehensive DHFA Indicator Taskforce database in the present study may have influenced the relative contribution of individual predictors within the prediction model.

Compared to the limited number of recent studies that have identified predictors of discharge to geriatric rehabilitation or inpatient care facilities [12,13,14], the present study focused on early predictors available immediately after surgery. Although several predictors reported in previous studies were included, some potentially relevant variables were not available in the DHFA Indicator Taskforce database. In particular, multiple comorbidities (diabetes [12,13], congestive heart failure [12], chronic renal failure [12], history of cerebrovascular accident [12], hypertension [12,13], chronic obstructive pulmonary disease [12,13], baseline anticoagulation therapy [12], and history of myocardial infarction [12]) which have previously been associated with discharge to geriatric rehabilitation or inpatient care facilities, may have contributed to improved model performance.

Patients with a history of dementia were less likely to be discharged to geriatric rehabilitation (vs. (nursing) home), which is consistent with current clinical practice, in which cognitive impairment is typically considered a contraindication for geriatric rehabilitation. Furthermore, these patients were more likely to be discharged to a nursing home rather than home, suggesting that cognitive impairment influences discharge planning toward settings that can provide long-term care. Nevertheless, some patients with a history of dementia were still discharged home. This likely reflects the availability of non-professional help, adequate home support from community services, and the patient’s functional independence. These variables, however, were not available in the DHFA Indicator Taskforce database.

The limitation of this study is that the DHFA Indicator Taskforce did not include variables related to the patient’s social context. Although the inclusion of several new variables led to a modest improvement in the predictive performance of the model, it remains suboptimal. To strengthen its predictive power, it is essential to include additional variables such as comorbidities and social context (for example, availability of non-professional help) [4,12,13]. By combining the predictors identified in recent studies with those found in the current study into a single, comprehensive dataset and repeating the analysis, it would be possible to assess which variables most strongly influence discharge destination. Future research should therefore prioritize collecting this information. In addition, a recent study with geriatric hip fracture patients has shown that higher fatigue resistance and grip work at hospital admission are associated with better functional recovery [18]. Incorporating these variables into the model may improve the timely identification of discharge destination. Furthermore, several postoperative factors may be associated with discharge destination. For example, early postoperative mobilization has been identified in the literature as a predictor of an increased likelihood of discharge home [19]. However, the objective of the current study was to support early discharge planning immediately after surgery. As mobilization data are generally not available until the first postoperative physiotherapy session one day after surgery, these variables could not be included. Future research should evaluate whether the addition of early postoperative mobilization (if available) improves the predictive performance enough to justify the later timing of the prediction model. In addition, future studies could explore destination-specific prediction models, in which each discharge destination is evaluated separately against all other destinations combined. Although our multinomial approach better reflects the clinical decision-making process involving multiple discharge options, destination-specific models may provide additional insight into the impact of predictor variables on specific discharge destinations. Ultimately, the goal remains to develop a strong discharge destination prediction score that predicts the discharge destination directly after hip fracture surgery.

## 5. Conclusions

This study identified several independent early predictors of discharge destination in patients aged ≥70 years following hip fracture surgery. These included age, gender, PFMS, premorbid Katz-ADL, ASA score, polypharmacy, history of dementia, delirium during previous hospital admissions, fall in the past six months, surgical treatment, anaesthesia type, and cotreatment by a geriatrician. Associations between these variables and discharge destination varied across the three pairwise comparisons. Discharge destination could be predicted immediately after surgery, with particularly high sensitivity for geriatric rehabilitation (86.7%), whereas prediction of discharge home and discharge to a nursing home remained limited (37.9% and 9.6%). The current performance makes the model not yet suitable for clinical use. Future research should therefore aim to refine the model by incorporating additional variables such as comorbidities and social context before clinical implementation can be considered.

## Figures and Tables

**Table 1:** Baseline and perioperative characteristics of the patients in the training set, presented for the total group and separately for each discharge group.

	Total (*n* = 2878)	Home (*n* = 991)	Rehabilitation (*n* = 1635)	Nursing Home (*n* = 252)	*p*-Value
Baseline variables					
Age in years; median (IQR *)	83.0 (77.0–89.0)	80.0 (74.0–86.0)	84.0 (79.0–89.0)	86.0 (81.3–90.0)	<0.001
Female gender; *n* (%) ^a^	2012 (70.0)	659 (66.7)	1169 (71.6)	184 (73.0)	0.017
Pre-fracture mobility score; *n* (%) ^b^					<0.001
- Fully mobile (1)	1293 (46.9)	583 (61.0)	645 (41.0)	65 (28.8)	
- Mobile with aids (2–3)	1084 (39.3)	271 (28.4)	693 (44.0)	120 (53.1)	
- Indoor confined (4–5)	379 (13.8)	101 (10.6)	237 (15.0)	41 (18.1)	
Premorbid Katz-ADL *; median (IQR *) ^c^	0 (0–2)	0 (0–1)	0 (0–2)	2 (1–5)	<0.001
ASA * score; *n* (%) ^d^					<0.001
- 1–2	1176 (41.2)	513 (52.2)	597 (36.7)	66 (26.8)	
- 3–5	1678 (58.8)	469 (47.8)	1029 (63.3)	180 (73.2)	
Use of osteoporosis medication; *n* (%) ^e^	428 (17.6)	131 (14.2)	244 (18.7)	53 (26.0)	<0.001
Polypharmacy (>5 medications); *n* (%) ^f^	1521 (54.2)	413 (43.1)	944 (58.6)	164 (70.1)	<0.001
History of dementia; *n* (%) ^g^	368 (13.1)	124 (12.8)	148 (9.3)	96 (40.2)	<0.001
Memory problems; *n* (%) ^h^	744 (26.4)	216 (22.3)	389 (24.1)	139 (57.4)	<0.001
Delirium previous admission; *n* (%) ^i^	432 (16.1)	130 (14.1)	217 (14.1)	85 (38.5)	<0.001
Fall in past 6 months; *n* (%) ^j^	1489 (55.4)	412 (45.1)	915 (58.9)	162 (73.6)	<0.001
Malnutrition, *n* (%) ^k^	279 (9.9)	60 (6.3)	183 (11.4)	36 (14.9)	<0.001
Perioperative variables					
Serum Hemoglobin level; mean (SD *) ^l^	7.8 (1.0)	7.9 (1.0)	7.8 (1.0)	7.7 (1.1)	<0.001
Fracture type; *n* (%) ^m^					<0.001
- Collum fracture	1627 (57.4)	648 (66.1)	851 (52.7)	128 (53.3)	
- Trochanteric fracture	1142 (40.3)	314 (32.0)	723 (44.8)	105 (43.8)	
- Subtrochanteric fracture	65 (2.3)	18 (1.8)	40 (2.5)	7 (2.9)	
Surgical treatment; *n* (%)					<0.001
- Hemiarthroplasty	1204 (41.8)	413 (41.7)	687 (42.0)	104 (41.3)	
- Intramedullary implant	1179 (41.0)	319 (32.2)	743 (45.4)	117 (46.4)	
- Total hip arthroplasty	124 (4.3)	86 (8.7)	35 (2.1)	3 (1.2)	
- Sliding hip screw/cannulated screw	371 (12.9)	173 (17.5)	170 (10.4)	28 (11.1)	
Anaesthesia type; *n* (%) ^n^					0.015
- General anaesthesia	1042 (38.2)	361 (37.5)	571 (37.3)	110 (47.0)	
- Spinal anaesthesia	1687 (61.8)	602 (62.5)	961 (62.7)	124 (53.0)	
Cotreatment geriatrician; *n* (%) ^o^	2493 (86.8)	810 (82.1)	1455 (89.0)	228 (90.5)	<0.001

* ASA, American Society of Anaesthesiologists physical status classification; IQR, interquartile range; Katz-ADL, Katz index of independence in Activities of Daily Living; SD, standard deviation. ^a^ Number of missing = 5. ^b^ Number of missing = 122. ^c^ Number of missing = 108. ^d^ Number of missing = 24. ^e^ Number of missing = 449. ^f^ Number of missing = 73. ^g^ Number of missing = 73. ^h^ Number of missing = 55. ^i^ Number of missing = 197. ^j^ Number of missing = 192. ^k^ Number of missing = 72. ^l^ Number of missing = 36. ^m^ Number of missing = 44. ^n^ Number of missing = 149. ^o^ Number of missing = 5.

**Table 2:** The final multinomial regression model.

	Discharge Geriatric Rehabilitation vs. Home ^a^			Discharge Geriatric Rehabilitation vs. Nursing Home ^a^			Discharge Nursing Home vs. Home ^a^		
	OR	95% CI	*p*-Value	OR	95% CI	*p*-Value	OR	95% CI	*p*-Value
Baseline variables									
Age	1.1	1.0–1.1	<0.001	1.0	1.0–1.0	0.999	1.1	1.0–1.1	<0.001
Gender									
- Female ^b^	--	--	--	--	--	--	--	--	--
- Male	0.8	0.6–1.0	0.029	1.1	0.7–1.7	0.606	0.7	0.5–1.1	0.113
Pre-fracture mobility score									
- Fully mobile (1) ^b^	--	--	--	--	--	--	--	--	--
- Mobile with aids (2–3)	1.8	1.4–2.2	<0.001	0.9	0.6–1.3	0.488	2.1	1.3–3.2	0.002
- Indoor confined (4–5)	2.1	1.5–3.0	<0.001	1.0	0.5–1.7	0.867	2.2	1.2–4.0	0.010
Premorbid Katz-ADL *	1.0	0.9–1.0	0.303	0.8	0.7–0.9	<0.001	1.2	1.1–1.3	<0.001
ASA * score									
- 1–2 ^b^	--	--	--	--	--	--	--	--	--
- 3–4	1.4	1.1–1.7	0.005	1.1	0.7–1.6	0.769	1.3	0.8–2.0	0.262
Polypharmacy (>5 medications)									
- No ^b^	--	--	--	--	--	--	--	--	--
- Yes	1.5	1.2–1.9	<0.001	0.7	0.5–1.1	0.137	2.1	1.4–3.1	<0.001
History of dementia									
- No ^b^	--	--	--	--	--	--	--	--	--
- Yes	0.6	0.4–0.8	0.002	0.3	0.2–0.4	<0.001	2.0	1.2–3.2	0.005
Delirium previous admission									
- No ^b^	--	--	--	--	--	--	--	--	--
- Yes	0.7	0.5–0.9	0.014	0.6	0.4–0.9	0.017	1.1	0.7–1.8	0.605
Fall in past 6 months									
- No ^b^	--	--	--	--	--	--	--	--	--
- Yes	1.5	1.2–1.8	<0.001	0.9	0.6–1.4	0.685	1.6	1.1–2.5	0.018
Perioperative variables									
Surgical treatment									
- Hemiarthroplasty ^b^	--	--	--	--	--	--	--	--	--
- Intramedullary implant	1.6	1.3–2.0	<0.001	1.1	0.8–1.6	0.640	1.5	1.0–2.2	0.053
- Total hip arthroplasty	0.4	0.2–0.7	<0.001	1.1	0.2–4.9	0.911	0.4	0.1–1.7	0.196
- Sliding hip screw/cannulated screw	0.8	0.6–1.0	0.069	0.9	0.5–1.6	0.675	0.9	0.5–1.6	0.628
Anaesthesia type									
- General anaesthesia ^b^	--	--	--	--	--	--	--	--	--
- Spinal anaesthesia	0.9	0.7–1.1	0.320	1.9	1.3–2.7	<0.001	0.5	0.3–0.7	<0.001
Cotreatment geriatrician									
- No ^b^	--	--	--	--	--	--	--	--	--
- Yes	1.4	1.0–1.8	0.045	0.5	0.2–0.9	0.032	2.9	1.4–5.7	0.003

* ASA, American Society of Anaesthesiologists physical status classification; Katz-ADL, Katz index of independence in Activities of Daily Living. ^a^ Data from a total of 2227 patients were used to build the multinomial regression model. ^b^ This parameter is set as a reference category.

**Table 3:** Classification table of the multinomial regression model for the test set and its performance.

Predicted Discharge Destination:	Observed Discharge Destination:		
	Home	Rehabilitation	Nursing Home ^a^
Home	95	56	5
Rehabilitation	149	383	42
Nursing home	7	3	5
AUC ^b^ (95% CI)	0.708 (0.668–0.748)	0.690 (0.651–0.728)	0.737 (0.667–0.807)
Sensitivity (95% CI)	0.379 (0.321–0.440)	0.867 (0.832–0.895)	0.096 (0.042–0.206)
Specificity (95% CI)	0.877 (0.845–0.903)	0.370 (0.317–0.425)	0.986 (0.974–0.992)
Precision (95% CI)	0.609 (0.531–0.682)	0.667 (0.628–0.705)	0.333 (0.152–0.582)
Prevalence (95% CI)	0.337 (0.304–0.372)	0.593 (0.558–0.628)	0.070 (0.054–0.090)
Detection prevalence (95% CI)	0.209 (0.182–0.240)	0.771 (0.739–0.799)	0.020 (0.012–0.033)
Balanced accuracy (95% CI)	0.628 (0.143–0.945)	0.618 (0.139–0.942)	0.541 (0.109–0.919)
Overall accuracy (95% CI)	0.648 (0.613–0.683)		

^a^ This subgroup was underpowered (*n* = 15). Therefore, results for this group should be interpreted with caution. ^b^ AUC: area under the curve.

## Data Availability

Restrictions apply to the availability of these data. Data were obtained from the Dutch Institute for Clinical Auditing (DICA) and are available from https://dica.nl/data-aanvragen/ (accessed on 8 September 2026) with the permission of the Dutch Hip Fracture Audit (DHFA) scientific committee.
